# Long-term effects of infrapatellar fat pad SVF infiltration in knee osteoarthritis management: A prospective cohort study

**DOI:** 10.1016/j.bonr.2025.101827

**Published:** 2025-01-24

**Authors:** Klaus Werner Labarre, Gerald Zimmermann

**Affiliations:** Department of Trauma Surgery and Sports Traumatology, Brothers' Hospital Julia Lanz Mannheim, Bassermannstraße 1, 68165 Mannheim, Germany

**Keywords:** Knee osteoarthritis, Stromal vascular fraction, Infrapatellar fat pad, Pain management, Functional improvement, Regenerative medicine

## Abstract

**Background:**

Knee osteoarthritis (OA) is a prevalent and debilitating condition that significantly impacts patients' quality of life and poses a substantial socioeconomic burden. Current treatments, including nonsteroidal anti-inflammatory drugs (NSAIDs) and physical therapy, often provide only temporary relief and fail to halt disease progression, particularly in advanced stages where knee replacement surgery becomes the primary option. Regenerative cell therapies, particularly those utilizing mesenchymal stem cells (MSCs), have emerged as promising alternatives due to their anti-inflammatory and regenerative properties. This study investigates the efficacy of stromal vascular fraction (SVF) derived from autologous adipose tissue when injected into the infrapatellar (Hoffa's) fat pad, an approach that leverages the rich vascular and stem cell environment of the fat pad to potentially modulate inflammation and promote tissue repair.

**Methods:**

Patients receiving therapy with SVF were invited to participate in the study. Inclusion criteria encompassed male and female patients aged 18 years or older with a Kellgren-Lawrence score up to 4, while exclusion criteria included malignant tumors, sepsis, or skin lesions at the site of collection or injection. A total of 25 patients were included in the study cohort, with two patients receiving bilateral treatment, resulting in 27 knees analyzed.

For the correlation analysis, an additional four patients who had only completed the six-month follow-up were included, one of whom underwent bilateral treatment. This extended the correlation analysis cohort to 29 patients and 32 knees. However, these four patients were excluded from the final study analysis as they had not completed the two-year follow-up. Consequently, the final analysis focused exclusively on the 25 patients (27 knees) who completed the full two-year follow-up.

**Results:**

Significant improvements were observed in VAS pain scores and KOOS subscales for pain, activities of daily living (ADL), and quality of life (QOL) at 6 and 24 months (*p* < 0.05). The correlation between the number of injected cells and functional improvements was significant for ADL at 6 months (Spearman's rho = 0.31, *p* = 0.044). This time point was prioritized to evaluate early therapeutic responses, as it represents a critical window when cellular activity and therapeutic effects are believed to peak. Focusing on the six-month follow-up allowed for a detailed assessment of these early impacts while minimizing potential confounding factors observed in later stages. No major complications were reported.

**Conclusion:**

SVF infiltration into the infrapatellar fat pad shows promising long-term benefits in pain relief and functional improvement for knee OA patients. Despite the lack of blinding and a control group, these findings suggest that SVF therapy could be a viable minimally invasive alternative to more invasive surgical interventions.

## 1 Introduction

Knee osteoarthritis is a leading cause of disability and a significant socioeconomic burden worldwide. Its prevalence is closely linked to an aging population, highlighting the urgent need for effective management strategie (Brooks, 2002; Fransen et al., 2011; Issa and Sharma, 2006). While various traditional treatments such as NSAIDs and physical therapy exist, they often fail to halt disease progression, especially in advanced stages where knee replacement surgery frequently remains the only viable option. Conventional treatments for knee osteoarthritis predominantly aim at managing symptoms rather than addressing the underlying causes. While these standard interventions may offer some degree of symptomatic relief, they are often accompanied by adverse side effects that can detract from their overall utility (Abraham et al., 2007; Bagga et al., 2006; Hawker et al., 2011; McAlindon et al., 2014).

To effectively address the treatment of osteoarthritis, a comprehensive understanding of its causes and pathophysiology is imperative. Despite ongoing research, the complete elucidation of these factors remains elusive, with current knowledge suggesting that osteoarthritis is a multifaceted disorder. It is characterized by a complex interplay of mechanical joint issues and genetic factors, which contribute variably to its onset and progression. Furthermore, emerging evidence strongly implicates inflammatory processes as key drivers in the advancement of the disease (Nehrer and Neubauer, 2021; Loeser et al., 2012).

As regenerative cell therapies continue to evolve, the therapeutic potential of cell-based approaches, particularly for degenerative conditions like osteoarthritis, is becoming increasingly recognized. Mesenchymal stem cells (MSCs) possess significant anti-inflammatory properties, which not only alleviate symptoms but also potentially modify disease progression by influencing the inflammatory processes central to osteoarthritis pathology.

Mesenchymal stem cells (MSCs), derived from the mesodermal layer, are renowned for their multipotent capabilities and their potential to differentiate into various cell types, including osteoblasts, chondroblasts, chondrocytes, myocytes, and adipocytes (Caplan, 2007). The precise mechanisms by which MSCs facilitate cartilage regeneration remain to be fully elucidated. However, it is hypothesized that their integration into the host tissue, support of growth and differentiation, and the beneficial effects of their secretions play pivotal roles in this regenerative process (Zwolanek et al., 2017).

Once administered, MSCs are believed to actively migrate to specific target tissues through a phenomenon known as the “homing” effect, which is mediated by their interaction with various chemokine receptors (Khaldoyanidi, 2008; Sohni and Verfaillie, 2013). In preclinical research, it has been observed that when mesenchymal stem cells (MSCs) are injected directly into the joint space, they preferentially target areas of cartilage damage and become incorporated into the synovial membrane, thereby initiating a healing process (Park et al., 2017; Mizuno et al., 2008; Kouroupis et al., 2019). It appears that the attachment of MSCs to damaged cartilage primarily facilitates the orchestration of regeneration, rather than their direct differentiation into new chondrocyte (Zwolanek et al., 2017; de Windt et al., 2017).

MSCs are known to secrete a wide array of bioactive factors that fall into three main categories: growth factors, cytokines, and extracellular vesicles (Arslan et al., 2013; Lai et al., 2010; Lai et al., 2013). Notably, they secrete anti-inflammatory cytokines such as hypoxia-inducible factor (HIF) and insulin-like growth factor-1 (IGF-1), which are thought to respectively foster chondrogenesis and enhance MSC proliferation and differentiation (Cantinieaux et al., 2013; Amann et al., 2017; Lee et al., 2011). Additionally, MSCs are known to safeguard chondrocytes from apoptosis by upregulating anti-apoptotic proteins and downregulating proapoptotic factors (Puetzer et al., 2010; Shang et al., 2014). The extracellular vesicles they release also play a role in cartilage repair, possibly through mechanisms akin to paracrine signaling (Rani et al., 2015; Tan et al., 2021). The collective action of these factors produced by MSCs is believed to synergistically contribute to the regeneration of cartilage tissue.

Pain alleviation and functional enhancement have been documented in preclinical animal studies following intra-articular MSC injections into arthritic joints, findings that are echoed in clinical settings where MSCs have been employed to treat knee joint arthrosis (Kouroupis et al., 2019; Jo et al., 2014; Murphy et al., 2003; Lee et al., 2007). Early-phase clinical trials have reported a reduction in cartilage defects, confirmed by radiological and arthroscopic evaluations, after patients received varying doses of in vitro expanded MSCs (Freitag et al., 2019; Lu et al., 2019).

MSCs can be sourced from various bodily tissues, typically bone marrow or adipose tissue. There is a distinction between adipose-derived stem cells (ADSCs) and bone marrow-derived stem cells (BMSCs), though they share similar pathophysiological properties. In line with the criteria set by the International Society for Cellular Therapy (ISCT) in 2006, MSCs should be plastic adherent, express markers like CD105, CD73, and CD90, and lack markers such as CD45 and CD34. Additionally, they should possess the capability to differentiate into cell types including osteoblasts, adipocytes, and chondroblasts (Dominici et al., 2006).

In our study, we opted for using stromal-vascular fraction (SVF) over traditional, culture-expanded mesenchymal stem cells (MSCs) due to several compelling advantages. Cultivation and in vitro expansion of MSCs, while yielding a high number of nucleated cells rich in MSCs, is time-intensive and cost-prohibitive (Baxter et al., 2004). More critically, this method may compromise the innate ‘homing’ ability of the cells a key mechanism by which MSCs localize to areas of tissue damage (Glowacki et al., 2015; Khaldoyanidi, 2008; Sohni and Verfaillie, 2013). There is also the associated risk of cellular senescence, telomere shortening, and phenotypic alterations, factors that could potentially diminish the therapeutic effectiveness of the cells (Baxter et al., 2004). Furthermore, culture-expanded cells are regulated as Advanced Therapy Medicinal Products (ATMPs) in Europe, subject to stringent regulatory controls whereas SVF can be isolated and applied with minimal manipulation, not falling under the initial ATMP classification (rev.1 E, 2015). This distinction eases the regulatory burden significantly, though regional approval is still requisite. SVF offers a heterogeneous population of regenerative cells, including ADSCs, macrophages, blood cells, pericytes, fibroblasts, endothelial cells, smooth muscle cells, and their progenitors, which collectively may contribute to the regenerative process (Bourin et al., 2013). While the concentration of MSCs in SVF may be variable and generally lower than that found in cultured ADSCs, it does not necessarily correlate with a reduction in clinical efficacy. Indeed, the safety and efficacy of SVF cells have been substantiated across various medical disciplines, showing promise in early studies for the treatment of knee osteoarthritis through intra-articular injections (Alatab et al., 2019; Lasso et al., 2018; Borakati et al., 2018; Di Matteo et al., 2019).

The infrapatellar fat pad (IFP) presents an optimal site for SVF therapy due to several anatomical and biological characteristics. Positioned within the knee joint, the IFP is a highly vascularized structure that harbors a rich supply of mesenchymal stem cells (Wickham et al., 2003; Bravo et al., 2018). Moreover, the IFP has been identified as an active participant in the pathophysiology of knee osteoarthritis, involved in the inflammatory processes that characterize the disease (Belluzzi et al., 2019; Klein-Wieringa et al., 2011; Greif et al., 2020). By directly infiltrating the IFP with SVF, there is a dual advantage: the local delivery of a concentrated mixture of regenerative cells, including ADSCs, and an immediate interaction with the joint's inflammatory environment (Lapuente et al., 2020). This strategic positioning allows for the potential exploitation of the SVF's anti-inflammatory and regenerative capabilities, which may result in modulating the local inflammatory milieu and promoting tissue repair.

Furthermore, the IFP is recognized as a reservoir for cytokines and growth factors that play a role in joint homeostasis and pathology, suggesting that SVF cells deployed in this environment could participate in a biologically synergistic process of repair and regeneration (Lapuente et al., 2020). The inherent proximity of the IFP to the synovial membrane and cartilage also implies that the injected cells are well-placed to exert their effects on the target tissues affected by osteoarthritis, capitalizing on the SVF's inherent “homing” properties (Macchi et al., 2018; Eymard et al., 2014). Given these points, along with the pragmatic aspect of regulatory compliance within the European context that permits SVF injections specifically in the Hoffa's fat pad, the IFP emerges as a strategic and logical choice for SVF therapy in our study.

Our preceding study delved into the feasibility and safety of autologous SVF infiltration into the Hoffa's fat pad for patients with knee osteoarthritis. The findings indicated significant pain relief and functional improvement over a one-year follow-up, demonstrating the promising potential of this approach (Labarre and Zimmermann, 2022). Building upon these results, this paper seeks to further investigate the long-term efficacy and clinical implications of SVF therapy. We hypothesize that continuous SVF treatment could provide a viable, minimally invasive alternative for managing knee osteoarthritis, with the possibility of delaying or even circumventing the need for more invasive surgical interventions.

This study offers an exhaustive analysis of two-year follow-up data, focusing on the durability of therapeutic benefits, the impact on patient quality of life, and the safety profile of SVF infiltration into the Hoffa's fat pad. Through this research, we aim to contribute significant insights to the evolving domain of osteoarthritis treatment and highlight the potential role of regenerative medicine in tackling this widespread and debilitating condition.

## 2 Materials and methods

### 2.1 Study design

This is a prospective single-center study to evaluate the efficacy of SVF therapy in patients with knee osteoarthritis. All described human studies have been conducted with the approval of the responsible Ethics Committee, in accordance with national law, and in accordance with the Declaration of Helsinki of 1975 (in the current, revised version). The therapy has been approved by the responsible regional council. A declaration of consent has been obtained from all patients involved.

### 2.2 Clinical outcome

The Visual Analog Scale (VAS) and the Knee Injury and Osteoarthritis Outcome Score (KOOS) were used as primary measures for a comprehensive assessment of patients' knee health and overall well-being. Commencing on the day of treatment, participants were required to complete the Visual Analog Scale (VAS) and the Knee injury and Osteoarthritis Outcome Score (KOOS) to establish baseline data for symptomatic and functional status. Thereafter, follow-up VAS questionnaires were disseminated at two weeks, six weeks, three months, six months, 12 months, and 24 months post-treatment, while the KOOS was reassessed at the three-month mark and subsequently at six months, 12 months, and 24 months. These surveys were distributed electronically via the Research Electronic Data Capture (REDCap) system, a secure web application for building and managing online surveys and databases, which facilitated the efficient and reliable collection of longitudinal data via email. This strategic approach allowed for a comprehensive analysis of temporal patterns in patient-reported outcomes, thereby illuminating the long-term efficacy and recovery trajectories associated with the treatment.

#### 2.2.1 Visual analog scale (VAS)

VAS is an instrument for measuring subjective pain intensity. Here, patients enter their pain on a vertical line. The ends of this line represent extreme values. Left: “no pain” and right: “extreme pain”. The given values are quantified with points from 1 to 10 (Thong et al., 2018).

#### 2.2.2 Knee injury and osteoarthritis score” (KOOS)

The Knee Injury and Osteoarthritis Outcome Score (KOOS) is a validated tool used to evaluate clinical limitations in patients with knee joint osteoarthritis. The KOOS consists of five subscales:

• Pain (pain)
• Symptoms
• activities of daily living (ADL)
• Function in sport and recreation Sport/Rec
• Quality of life related to the affected knee (knee related quality of life - QOL)

Patients are required to answer 42 questions at specified intervals. Each question is assigned a point value, with a total possible score ranging from 0 to 100. A score of 100 points indicates almost no restrictions, whereas a score of 0 points indicates the maximum possible restrictions (Collins et al., 2016).

### 2.3 Protocol for cell preparation, viability testing, and injection

Using the Arthrex ACA Kit® (Arthrex GmbH, Naples, FL, USA), 30 ml of lipoaspirate was collected from the lower abdomen in two Arthrex ACP® double syringes. A Carraway Harvester® (Tulip Medical Products, San Diego, CA, USA) was connected to the syringes. The Arthrex ACP® double syringe consists of a large and a small syringe, which is located in the plunger of the large syringe. The small syringe can be used to remove a liquid fraction that is above a solution after centrifugation without contaminating the remaining or the removed product.

**Fig. 1:**
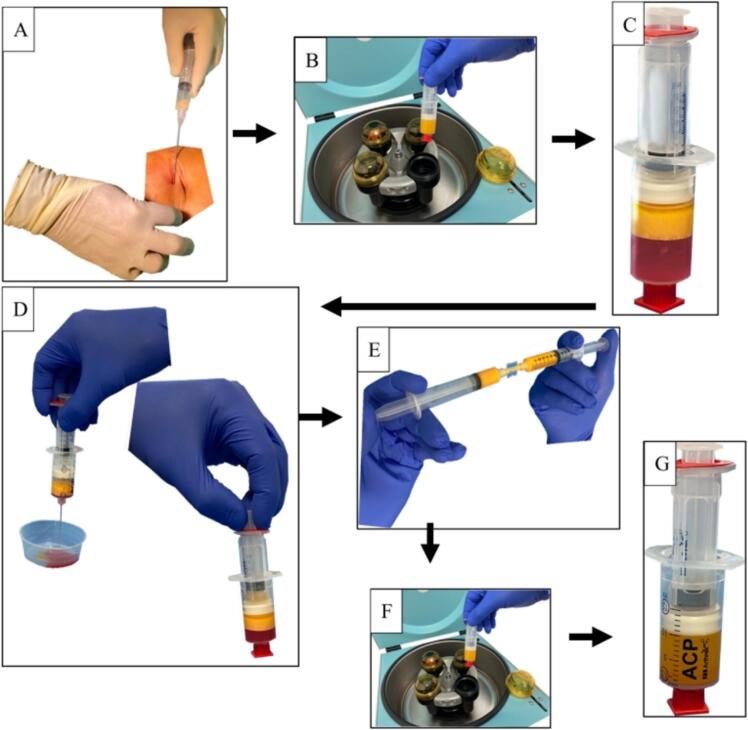
(A) Removal of the fat tissue, (B) Centrifugation of the fat tissue at 2500 rpm for 4 min (C) Lipoaspirate after the first centrifugation with aqueous fraction at the bottom, the fat graft in the middle and an oil layer on top. (D) The aqueous fraction is removed and the oil is transferred to the small syringe and also removed. (E) The fat graft was transferred into two 10 ml Luer lock syringes and then transferred from one syringe to the other at least 30 times for homogenization using a 1.4 mm connector. (F) Second centrifugation at 2500 rpm for 4 min. (G) After the second centrifugation, there is an SVF pallet of about 1 ml at the bottom of the double syringe. Syringe with an oil layer from the destroyed adipocytes on top (Labarre and Zimmermann, 2022).

The lipoaspirate, divided into 15 ml portions per double syringe, was centrifuged at 2500 rpm in a centrifuge (Rotofix 32A® (Andreas Hettich GmbH & Co. KG, Tuttlingen, Germany)) for 4 min at room temperature (Fig. 1B). The lipoaspirate was divided into oil, fat graft, and an aqueous fraction (Fig. 1C). The oil was transferred to the small syringe and discarded. The aqueous fraction was removed (Fig. 1D). The fat graft was transferred into two 10 ml Luer-Lock syringes and then transferred at least 30 times from one syringe to the other for homogenization using a 1.4 mm connector (Fig. 1E).

20 ml of fat graft per collection could be isolated and transferred 30 times from one syringe to the other for further processing. The fat graft was then centrifuged again at 2500 rpm for 4 min. Afterwards, a pellet of 1 ml in size containing the SVF was visible at the bottom of the syringe. Above it was a layer of oil from the destroyed adipocytes, which was aspirated by the small syringe (Fig. 1G). For better application of the SVF, it was diluted with 5 ml NaCl 0.9 % solution. Before injection, a sample of SVF was taken to determine the number of nucleated cells and the proportion of vital cells.

The cell count was determined by using a NucleoCounter NC-200® cell counter (ChemoMetec A/S, Allerod, DK, Denmark). An untreated sample was filled into a Via 1 cassette, which stained nucleoli of dead cells with 4′,6-Diamidine-2-phenylindole (DAPI). The number of nucleoli of dead cells was determined by the cell counter. A second portion of the sample was treated with Reagent A100 and Reagent B, which led to lysis of the cell membranes. In the pretreated sample, all cells were stained with DAPI, and the total number of cells was determined by the cell counter. From the difference, the percentage of vital cells was calculated, which is automatically indicated by the cell counter. The injected volume was recorded for each patient. Thus, the number of actually injected cells and the percentage of vital cells could be calculated. The time between fat harvesting and cell analysis varied between 1 and 4 h while the samples were stored at room temperature.

The stromal-vascular fraction was injected into Hoffa's fatty body under sonographic control. The whole procedure, from fat extraction to injection, took about 1 h. Patients could be mobilized and discharged immediately after injection of the SVF. No further physiotherapeutic treatments or interventions were performed in the postoperative period.

### 2.4 Data analysis

R version 4.1.1 (2021-08-10) was used to perform the analyses and create the graphs. For the repeated measures, only patients who had completed the two-year follow-up were included. Missing values were replaced by “Multivariate Imputation by Chained Equations (MICE)”. The Friedman test was used to find statistically significant differences between the repeated measures of the subscales of the KOOS and the VAS. The Wilcoxon signed-rank test was used to examine differences between the individual follow-up time points. P-values were adjusted with the Bonferroni correction. To test for a correlation between clinical outcome and number of injected cells, the percentage change in clinical score at 6 months compared to baseline was correlated with the number of injected cells. Data were not normalized to cell count due to the heterogeneous patient population and the exploratory nature of the study. For this analysis, only patients who had completed the 6-month follow-up and for whom the number of injected cells was measured were included. Missing values were replaced by “Multivariate Imputation by Chained Equations (MICE)”. Spearman's rank correlation was used to test for significance.

## 3 Results

### 3.1 Patient characteristics

Patients receiving therapy with SVF were invited to participate in the study. Included were all male and female patients aged 18 years or older with a Kellgren-Lawrence score up to 4. Exclusion criteria included patients with malignant tumors, sepsis, or skin lesions at the site of collection or injection.

In total, 25 patients were included in the main study cohort. Notably, two of these patients received bilateral treatment, and to account for distinct outcomes for each knee, they were treated as separate entries in the analysis. This resulted in a total of 27 knees analyzed. The age of participants ranged from 53 to 67 years, with a median age of 61 years, representing the target demographic for the evaluated treatment. Regarding gender distribution, 44 % were female and 56 % were male.

For the correlation analysis, four additional patients who had only completed the six-month follow-up were included. Of these, one patient received bilateral treatment. This resulted in a total of 32 knees analyzed for the correlation analysis. Importantly, these four patients were not included in the final study analysis, as they had not completed the two-year follow-up.

The final analysis, therefore, focused exclusively on the 25 patients (27 knees) who completed the full two-year follow-up, distinguishing this group from the broader cohort utilized for the correlation analysis.

### 3.2 VAS and KOOS

**Fig. 2:**
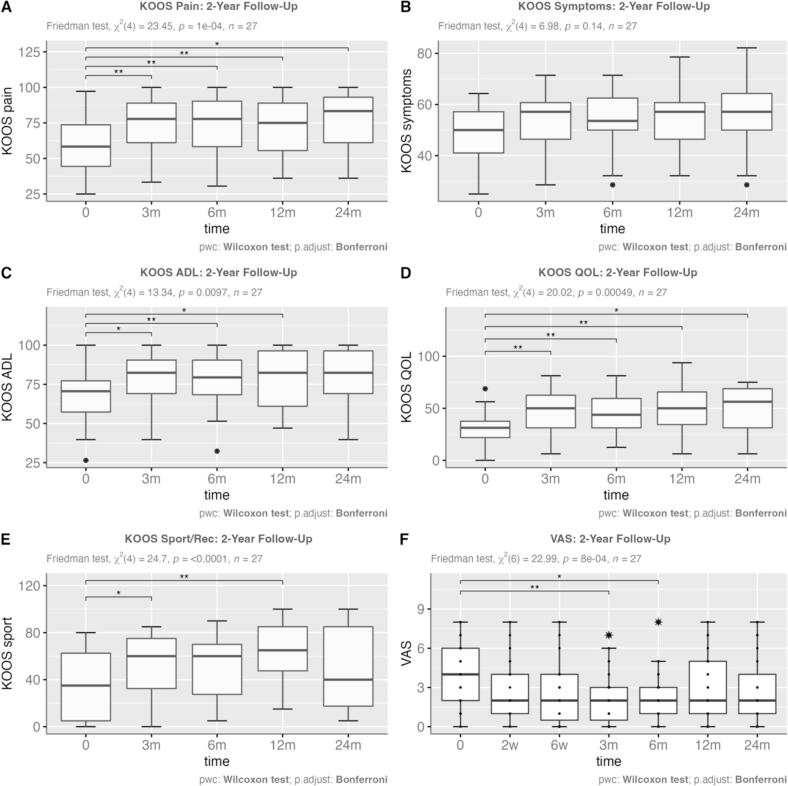
The figure shows the subscales of the KOOS before therapy and after 3, 6,12 and 24 months(A-E) as well as the VAS before therapy, after 2 weeks, 6 weeks, 3 months, 6 months 12 months and 24 months(F). Significances are marked with an * (*p.adj. < 0.05; ** p.adj. <0.01; *** p.adj. < 0.001;**** p.adj. < 0.0001) . Analysis includes data from a cohort of 27 patients.

Over the course of the two-year follow-up, the study demonstrated notable improvements in pain management and functional capabilities for patients with knee osteoarthritis, as detailed in Fig. 2. There was a significant reduction in KOOS pain scores at 6 and 24 months (*p* = 0.04, *n* = 27), suggesting enduring pain relief (Fig. 2A). The KOOS symptoms subscale did not show significant changes throughout the study period (*p* = 0.14, *n* = 27) (Fig. 2B). Activities of daily living (ADL), a crucial component of KOOS, improved significantly at 6 months and continued to show benefits up to 24 months (*p* = 0.0097, *n* = 27) (Fig. 2C). Quality of life (QOL) as assessed by KOOS saw significant enhancements, particularly at 6 and 12 months (*p* = 0.00049, n = 27) (Fig. 2D).

In the sports and recreational activities subscale of KOOS, the greatest improvements were observed at 6 months, with the effects persisting at subsequent assessments (*p* < 0.0001, n = 27) (Fig. 2E). Additionally, the VAS pain intensity scores showed significant improvement up to 6 months after therapy (*p* = 0.04, n = 27), but this improvement was not sustained after 12 and 24 months (Fig. 2F). In contrast, the KOOS pain subscale demonstrated sustained significant improvements at 12 and 24 months, highlighting a discrepancy between the tools in capturing long-term benefits of the therapy.

### 3.3 Cell count and correlation with clinical outcome

**Fig. 3:**
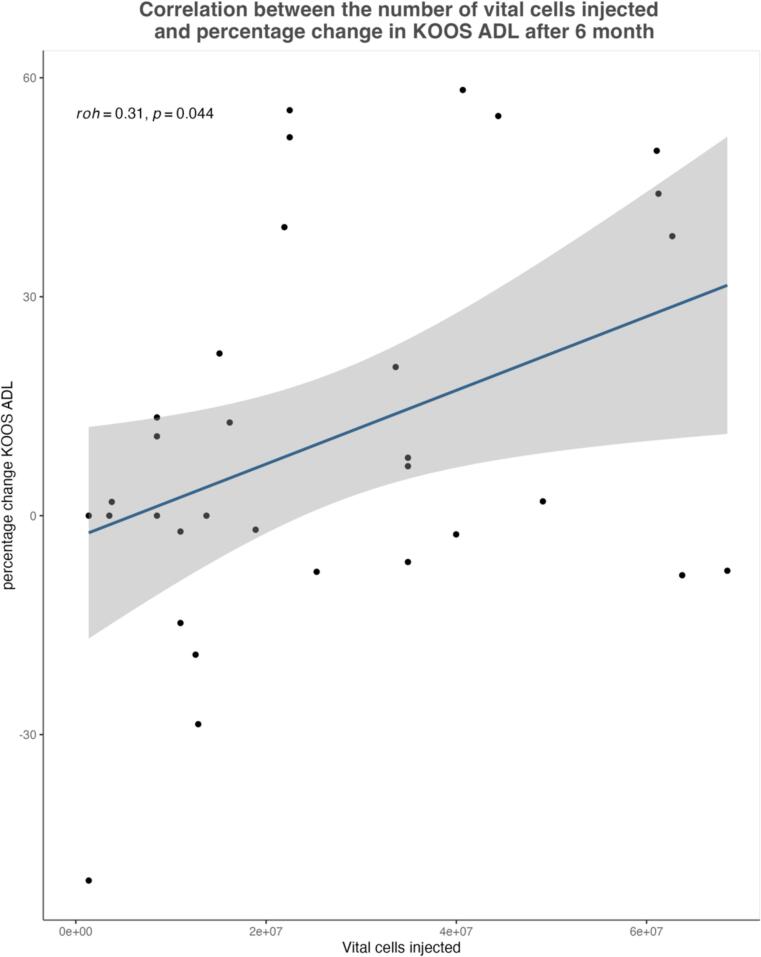
Shows the correlation between the number of vital injected cells and the percentage improvement after 6 months for the KOOS ADL subscale . The Spearman correlation coefficient was calculated. Analysis includes data from a cohort of 32 patients.

The median number of total cells injected per preparation was 33,420,000 (IQR: 34,797,500), while the median number of viable cells was 22,202,865 (IQR: 29,158,200). The correlation analysis between the number of viable cells injected and clinical improvement showed a significant positive correlation for the KOOS ADL subscale at 6 months (Spearman's rho = 0.31, *p* = 0.044). However, no statistically significant correlation was observed for the other KOOS subscales or the VAS pain score. (See Fig. 3.)

### 3.4 Complications

No major complications occurred in any of the patients.

## 4 Discussion

The approach we have chosen provides further prospective data using a technique that is consistent with existing European regulations. However, this study has notable limitations, including the relatively small sample size, the lack of a control group, possible bias due to self-reporting by patients, and the limited generalizability of findings due to the single-center design. We present a prospective case study investigating the clinical outcome after injection of SVF into Hoffa's fat pad as well as the cell count and viability of SVF prepared by mechanical processing of autologous adipose tissue. Previous studies on the use of SVF for the treatment of knee osteoarthritis are based on an enzymatic process to produce SVF and its intra-articular injection. In contrast, our method is based on a mechanical production process and the choice of IFP as the target tissue, for which only an approval for the homologous use of SVF is required in Germany. The aim of this pilot study was to evaluate the efficacy and therapeutic benefit of this new treatment method. This study included 25 patients, of whom 2 underwent bilateral treatment, resulting in a total of 27 knees analyzed for the two-year evaluation. Additionally, 4 further patients (including 1 with bilateral treatment) were included solely for the six-month follow-up correlation analysis, bringing the cohort for this analysis to 29 patients and 32 knees. These additional patients were not part of the final two-year evaluation, as they did not complete the full follow-up period. While the sample size is relatively small, it is adequate for a pilot study and serves as a foundation for establishing this method. The data generated here provide a first prospective evaluation of the method's feasibility, safety, and potential clinical benefits. Established and internationally recognized outcome scores were used to assess the long-term efficacy and clinical implications of SVF therapy. The study reports significant improvements in pain relief, functional enhancement, and quality of life for the patients, demonstrating the potential of this approach as a minimally invasive alternative for managing knee osteoarthritis.

The discrepancy between the VAS scores and the KOOS pain subscale reflects differences in their sensitivity to changes over time. While both the VAS and KOOS pain subscale showed significant improvements during the first 6 months, only the KOOS pain subscale maintained significance at later time points up to 24 months. This indicates that the KOOS pain subscale may better capture the sustained functional and symptomatic benefits of SVF therapy beyond early pain relief. The VAS, which measures subjective pain intensity, may be less responsive to long-term changes in overall pain experience compared to the KOOS pain subscale, which includes broader functional aspects. These findings underscore the importance of using complementary tools to assess both short- and long-term outcomes of regenerative therapies.

Due to the invasiveness and possible risks of fat removal, we did not include a placebo group for ethical reasons. However, it is important to acknowledge the potential influence of placebo effects in this study. The significant improvements reported by patients, particularly those based on subjective measures such as VAS and KOOS scores, may partly reflect expectations and psychological factors associated with undergoing a novel treatment. Future studies with appropriate control groups will be essential to differentiate the true therapeutic effects of SVF therapy from potential placebo responses. The number of injected cells showed high variance, primarily due to the different cell concentrations in one milliliter of injection solution and the total amount of starting material collected. In a recently published study from Japan, patients were injected with an SVF preparation that contained almost twice as many cells (76 million cells on average) compared to the preparation used here. In Japan, the Celution® 800/CRS system (Cytori Therapeutics Inc., San Diego, CA) was used. With 334.3 ± 44.0 ml, a significantly larger amount of lipoaspirate was obtained to produce SVF. For this comparatively more complex procedure, patients received general anesthesia. In the system used here, a maximum of 30 ml of lipoaspirate was harvested under tumescent anesthesia. As expected, treatment with SVF does not result in preparations with a constant cell count. In addition to technical and systematic differences, patient-related variables may also be decisive. To maximize the effect in patients, the entire amount of SVF was injected without adjusting the number of cells to a specific concentration after cell counting. However, we recognize that controlling and standardizing these parameters is essential for future studies to strengthen the reliability of the findings and to better understand the relationship between cell dose, viability, and therapeutic outcomes. The therapeutic benefit of different cell doses will be the subject of future follow-up studies with larger initial collection volumes. The viability of the injected cells also varied widely, with some showing comparatively low viability. This variability limits the ability to draw definitive conclusions about the efficacy of the procedure, as it introduces uncertainty regarding the consistency and reproducibility of the therapeutic effects observed. While the clinical improvements noted in the study are encouraging, further investigations with strict control of cell viability and standardized preparation protocols are essential to substantiate these findings. Further controlled studies with standardized cell viability measures, larger sample sizes, and the inclusion of a control group are required to validate these findings and mitigate the biases and limitations identified in this study. Further controlled studies with standardized cell viability measures and larger sample sizes are required to validate these findings and refine the procedure. However, this measurement is somewhat distorted by the fact that the time between cell collection and cell measurement varied between one and five hours, during which the preparation was stored at room temperature. The number of injected cells showed a clear correlation with the clinical results in the ADL and QOL sub4 scales of the KOOS. With this very heterogeneous patient population in terms of age and degree of osteoarthritis, it can be assumed that other factors besides the number and viability of the cells can influence the response to the therapy. However, since a significant correlation was found, it can be assumed that the number of injected cells and their viability are key influencing factors.

Several dose-escalation studies have found a clear association between higher cell doses and improved clinical outcomes and cartilage regeneration. For example, Freitag J. et al. (2019) demonstrated that 100E+06 ADSCs per injection yielded significant pain and function improvements at six months. In comparison, our study observed similarly significant improvements in pain and functional outcomes using mechanically processed SVF, though with a lower average number of cells and without in vitro expansion. However, our approach utilized mechanically processed SVF with varied cell viability and doses, which differ from the in vitro cultured ADSCs used in Freitag's study. Despite these differences, our results align with their findings, particularly in demonstrating meaningful pain relief and functional enhancement. However, our use of mechanically processed SVF with varied cell counts and viability highlights a potential advantage in terms of procedure simplicity and regulatory compliance, while also underscoring the need for further direct comparative studies to validate these observations. Further comparative analyses are required to establish how variations in cell preparation and dose affect clinical outcomes. There were no significant differences in pain and function between the one-injection group and the two-injection group even after one year. In the group with two injections, however, an improvement in cartilage quality could be demonstrated radiologically after one year, which was seen as an argument for greater therapeutic efficacy when injected again after 6 months. However, as the cell products used in the published studies differ greatly in terms of cell preparation, the values determined cannot be used for a general dose recommendation.

### 4.1 Conclusion

Our procedure represents a safe and minimally invasive option for the treatment of knee osteoarthritis. This pilot project highlights the potential of SVF therapy, but also underscores its limitations. The most important findings of our study include significant improvements in pain relief, functional enhancement, and quality of life observed over a two-year follow-up period. However, the comparatively low and widely varied viability of the injected cells limits the strength of our conclusions regarding the efficacy of the procedure. Compared to other procedures requiring general anesthesia or in vitro cell expansion, our method minimizes the effort and associated psychological and physical stress for patients. Our study indicates that the therapeutic effect can persist for up to two years. However, there is a possibility that its efficacy may diminish beyond this period. Further investigations with stricter controls on cell viability, precise dosing, and preparation protocols are required to confirm these findings and to establish whether repeating the therapy could sustain or even enhance the therapeutic outcomes. Current research on the optimal cell count remains limited. Nonetheless, we hypothesize that the quantity of cells administered falls within the therapeutically effective range. Our findings suggest that an increased cell count could enhance the therapeutic outcome.

### 4.2 Outlook

There are still many unanswered questions, and the effectiveness of this promising therapy can probably be increased considerably. Future studies should include a randomized controlled trial comparing low-dose versus high-dose SVF injections to evaluate dose-dependent efficacy. A randomized controlled trial with a placebo group would also be ideal to distinguish true therapeutic effects from psychological or expectation-related responses. However, given the invasive nature of the procedure, obtaining approval from ethics committees for such a study might be challenging. Innovative study designs will be needed to address these limitations while maintaining ethical standards. Additionally, comparisons with established treatments such as platelet-rich plasma (PRP) or hyaluronic acid injections are essential to determine the relative benefits of SVF therapy.

## CRediT authorship contribution statement

**Klaus Werner Labarre:** Writing – original draft, Supervision, Project administration, Methodology, Investigation, Funding acquisition, Formal analysis, Data curation, Conceptualization. **Gerald Zimmermann:** Writing – review & editing, Supervision, Funding acquisition, Conceptualization.

## Funding

This study was funded by grants provided to the Department of Trauma Surgery at Theresienkrankenhaus by the Karl Kärcher Foundation for research purposes. No additional funding was received.

## Declaration of competing interest

The authors declare the following financial interests/personal relationships which may be considered as potential competing interests:

Prof. Zimmermann has a consultancy agreement with Arthrex, whose double syringes are used for the production of SVF. No other conflicts of interest exist.

## Data Availability

Data will be made available on request.
